# Styles of Leadership, Fears of Compassion, and Competing to Avoid Inferiority

**DOI:** 10.3389/fpsyg.2018.02460

**Published:** 2019-01-22

**Authors:** Jaskaran Basran, Claudia Pires, Marcela Matos, Kirsten McEwan, Paul Gilbert

**Affiliations:** ^1^Centre for Compassion Research and Training, University of Derby, Derby, United Kingdom; ^2^Center for Research in Neuropsychology and Cognitive Behavioral Intervention (CINEICC), University of Coimbra, Coimbra, Portugal

**Keywords:** compassion, leadership style, competitive behavior, attachment, antisocial, prosocial

## Abstract

There is general agreement that styles of leadership evolved from mammalian group living strategies that form social ranks. In both non-human primates and humans, different styles of hierarchical dominant-subordinate and leader-follower behavior can be observed. These can be described in terms of dimensions of antisocial (relatively self-focused, aggressive and threat-based) and prosocial (relatively empathic, caring, and supportive) interpersonal styles. The aim of this study was to explore how a set of established self-report questionnaires might relate to these two dimensions. Two hundred and nineteen students completed questionnaires assessing ruthless self-advancement, coalition building, and dominant leadership styles, as well as hypercompetitiveness, narcissism, striving to avoid inferiority, compassion focused and ego focused goals, fears of compassion, social safeness and attachment (in)security. A principal component analysis supported an antisocial leadership style factor which comprised of ruthless self-advancement, narcissism and hypercompetitiveness. This was significantly correlated with fears of compassion, ego focused goals, insecure striving (striving to avoid inferiority), fears of losing out, fears of being overlooked, fears of being rejected, and avoidant relating in close relationships. It was significantly negatively correlated with compassionate goals. As the results did not reveal a clear factor solution for a prosocial leadership style, we chose to use the coalition building leadership style variable. This showed the opposite pattern, being significantly negatively correlated with narcissism, hypercompetitiveness, fears of compassion, fears of active rejection, and avoidance in close relationships. It was significantly positively correlated with secure striving, compassionate goals, and social safeness. We also found that fears of compassion for others was a partial mediator of the relationship between insecure striving with antisocial leadership style. Moreover, lower fears of compassion for the self emerged as a key mediator for the relationship between non-avoidant attachment with coalition building leadership style and, secure non-striving with coalition building leadership style. While the motive to accumulate social power, resources and dominance may be linked to antisocial forms of leadership, the intensity of the drive may also be linked to unaddressed threats and fears of rejection and fears of compassion. Efforts to promote more ethical, moral and prosocial forms of leadership may falter if such fears are left unaddressed.

## Introduction

Leadership styles tend to be referred to as the manner and approach people adopt when they are managing groups of people (Amanchukwu et al., 2015), but it can also relate to any relationship where there is a “leader and a follower(s)” (Gilbert, 1989/2016, 2018; Gilbert and Basran, 2018). This area has received notable attention. Meuser et al. (2016) reviewed 864 articles on leadership and identified over 40 different styles. However, several researchers have raised questions about the different models. In particular, concerns have been raised in relation to their distinctiveness, overlaps and similarities (Avolio and Gardner, 2005; Avolio and Walumbwa, 2014) and if the models (such as authentic leadership style) are only descriptive and do not articulate underlying processes, such as motivational and contextual factors, that give rise to specific styles (Cooper et al., 2005). For example, Van Knippenberg and Sitkin (2013) argue that the conceptual definition of charismatic–transformational leadership does not specify how different dimensions form charismatic–transformational leadership styles or how they are selected. In addition, there is an assumption that individuals develop these qualities through development of self-knowledge and self-awareness. Ford and Harding (2011) raise the issue that authentic leadership style models do not acknowledge the imperfections of individuals and the limitation on self-awareness.

A different approach to leadership style is to identify core dimensions that can be rooted in evolved algorithms and motives (Gilbert, 1989/2016, 2018; King et al., 2009; Gilbert and Basran, 2018). While exceptions exist, such as Humphrey et al.’s (2016) emotion-focused framework for leadership, and Van Vugt and colleagues’ exploration of leadership against the backdrop of the evolution of social hierarchies (Van Vugt et al., 2008; King et al., 2009), explorations into the evolutionary mechanisms underpinning leader-follower relationships require further consideration (Gilbert, 2018; Gilbert and Basran, 2018). Two dimensions underpinning different forms of competitive behavior and leadership style have been described as *antisocial* (relatively self-focused, threatening, and low on caring for others) versus *prosocial* (relatively other-focused, empathic, caring, and moral) (Gilbert, 1989/2016, 2018; King et al., 2009; Brañas-Garza et al., 2016; Ewest, 2017; Maner, 2017; Gilbert and Basran, 2018). Elsewhere, Gilbert and Basran (2018) have outlined how a range of survival and reproductive strategies can be mapped onto these dimensions. This article utilizes a range of currently available self-report scales to investigate the core attributes of these two dimensions.

### Antisocial Leadership Styles

At present, there are few agreed definitions to distinguish between antisocial and prosocial leadership styles (although see Ewest, 2017). We note, however, that there is a long history of studying antisocial behavior within criminological and psychopathological contexts. Features of antisocial disorders have been identified, such as callousness, aggressiveness, deceitfulness, lack of remorse, responding very aggressively to criticism – particularly from subordinates – and even enjoyment from making others suffer (e.g., see Piotrowska et al., 2015). Our use of the term is not to imply a specific personality disorder as such, but dimensional elements to social relating that can vary from person to person, contexts and in the blends of different traits. One way of defining an antisocial style is that it can be contrasted with its opposite, a prosocial, compassionate style. Compassion is typically defined “as a sensitivity to suffering in self and others with a commitment to try to alleviate and prevent it” (Gilbert, 2009; Gilbert and Choden, 2013; Gilbert et al., 2017). Generally, individuals seek to be helpful rather than harmful. Antisocial motivation and behavior can therefore be seen as an insensitivity to the suffering in self and others with a callous indifference or purposeful intent to cause it for one’s own interests (Gilbert, 2018). In the pursuit of self-advancement and power, those who adopt antisocial leadership styles can be indifferent to the needs and sensitivities of those they lead (unless they are manipulating others for their own self-interest), driven by threat, and tend to believe that respect depends on a degree of fear in subordinates (Scott, 1990).

History and modern-day boardroom politics are replete with examples of such leaders, who use threat and create environments of “walking on eggshells” around them (Scott, 1990; Lindholm, 1993), which create forms of insecure and destructive leadership styles (Schyns and Schilling, 2013). Leaders who adopt these styles may be seen as resentful, envious or fearful of subordinates doing well or taking credit and create a fearful and vigilant social context. They are more likely to explicitly or implicitly encourage infighting and competition within a group or team, especially for the leader’s favors (as did Hitler, Stalin, Jim Jones and many others) (Lindholm, 1993). Gilbert and Basran (2018) and other evolution theorists (e.g., King et al., 2009) suggest that antisocial leadership styles may be rooted in old, evolved strategies for resource and sexual competition.

A different approach to leadership styles, also rooted in evolutionary models of social competition, has been developed by Zuroff et al. (2010). They identified three different leadership styles: ruthless self-advancement, coalition building, and dominant leadership. From their self-report measure, dominant leadership items were based on dispositions to be dominant, assertive, and self-promoting to attain a leadership role. Coalition building items were based on building cooperative coalitions, consulting with others and seeking to compromise. The third dimension of ruthless self-advancement was based on advancing self-interests by any means, including those that may be unethical, deceptive, and disloyal to others around them. Studies suggest ruthless self-advancement is strongly associated with narcissism and negatively with agreeableness (Zuroff et al., 2010; Kelly et al., 2011). These items would seem to tap into antisocial leadership styles.

Antisocial leadership styles have also been linked to the dark triad of Machiavellianism, narcissism and psychopathy (Furnham et al., 2013). Among the other traits attributed to the dark triad are a sense of entitlement, superiority, social charm, impulsiveness, manipulativeness, lack of empathy, untrustworthy, brazenness, (over)confidence, lack of interest in the caring for others, callousness, poor handling of interpersonal conflict and criticism, poor insight into their own emotions, and shallowness (alexithymia). However, they can also be intelligent, persuasive, strategic and highly manipulative (Jonason and Krause, 2013). In their review of the dark triad, Muris et al. (2017) found these traits were more negatively correlated than previously thought with the personality dimensions of agreeableness and honesty-humility and were more prominent in men than women. They also noted that the way of measuring these traits is in its infancy, primarily focusing on self-reports, but that the study of the “malevolent side of human nature” as they call it (or antisocial style as we call it) is a key research concern, particularly because its impact can be so destructive.

As these are dimensions rather than categories, different combinations and types of traits and degrees of intensity will be contextually reflected in different styles. In the wider context, there is increasing evidence that in various schools and businesses, the focus is on the drive for a self-focused narcissistic competitive edge rather than community or compassionate values (Twenge et al., 2010; Sachs, 2012; Harvard Graduate School of Education, 2014; Music, 2014). Furthermore, research suggests that these leadership styles are seen more frequently the higher up in the organization the individual is and the more powerful the organizations are, partly because their ruthlessness can appear to be helpful to organizations and help them get on (Furnham et al., 2013). Indeed, around the world, it is not difficult to identify such characters in leadership positions today.

### Prosocial Leadership Styles

Whereas antisocial behavior evolved out of the evolutionary benefits from being able to coerce others and induce fearful submission, prosocial behavior evolved out of the benefits of caring, sharing, cooperation and being attractive as a sexual partner and ally; gaining what Barkow (1989) called prestige ranking. There is good archaeological evidence that early humans cared for each other such that even those with diseases and injuries survived (Spikins, 2017). There are a number of evolutionary theories that underpin the evolution of prosocial behavior such as the *social brain hypothesis* (Dunbar, 2016). This highlights the survival and reproductive benefits of altruistic behavior for buffering the stress of group living, reputation and status acquisition, and offering a variety of physiological and practical benefits (Gilbert, 2009). Leaders with a prosocial leadership style stand in direct contrast to those who adopt an antisocial leadership style. They have an interest in the development and wellbeing of others, are empathic to the needs, sensitivities and difficulties of those they lead, tend to be supportive and friendly (rather than threatening and unpredictable) and foster confidence and creativity (in contrast to fear and compliance or resistance) (Colonnello et al., 2017; Ewest, 2017). They can also be seen to endorse moral standards for themselves and their organization and try to avoid causing harm to themselves or others (Boyatzis et al., 2006). Ewest (2017) notes that prosocial leaders have a highly developed sense of responsibility. In attachment terms, they will try to create a sense of a secure base (Mikulincer and Shaver, 2007). In essence, they show a range of behaviors that have been associated with prosocial behavior (Penner et al., 2005) and compassionate behavior (Gilbert, 2009, 2018) in general. Prosocial leaders, be they parents, teachers or managers, have essentially a compassionate orientation (Bierhoff, 2005; Ricard, 2015; Gilbert et al., 2017; Seppälä et al., 2017).

de Zulueta (2015) points out that the quality of prosocial leaders depends on the context. For example, in health settings, leaders need to be able to be clinically competent and able to cope with the distress in their patients, in the staff who may be distressed working with them, and also themselves. This requires emotional engagement and regulation skills, plus abilities in shared forms of experiencing (rather than some heroic rescuer). West and Chowla (2017) and West et al. (2017) also indicate that prosocial leaders do many things for their team, such as promoting trust and mutual sharing and teambuilding, supporting creativity and innovation, and ensuring the team obtains the resources it needs to do the work. They can also enable individuals to have a sense of purpose and creativity as well as a sense of friendly belonging.

Nonetheless, what appears to be prosocial behavior and motivation can be complex and not always purely altruistic. For example, Böckler et al. (2016) meta-analyzed a number of self-report measures with behavioral indicators of prosocial behavior and suggested four types. Altruistic prosocial behavior was genuine helping and caring behavior with a cost to oneself. Norm-based prosocial behavior related to punishing unfair distribution of resources and was rooted in the norms of reciprocity and of “helping others when I’ve been helped.” Strategic prosocial behavior was based on cost-benefit calculations. Self-reported prosocial behavior was based on self-report measures that did not always correlate with behavioral measures. Catarino et al. (2014) explored what they called submissive compassion, which was behaving helpfully and compassionately in order to be liked and avoid rejection. These submissive forms of prosocial behavior may not be based on genuine empathic concerns for others, but rather on seeking to create a good reputation. Hence, one tries to be helpful without necessarily having empathic insight into what would be helpful. Unlike genuine compassion, submissive compassion was linked to depression and anxiety. These can also be seen as dimensions where individuals can do things for multiple reasons, allowing different combination of these motives in different contexts.

Given the increasing concern for leadership, businesses and politics to become more morally responsible and compassion focused (Boyatzis et al., 2006; Hannah et al., 2011; Sachs, 2012; Piketty and Ganser, 2014; Ewest, 2017; West et al., 2017; Worline and Dutton, 2017; Crimston et al., 2018), understanding the evolutionary and social roots of compassionate, ethical and prosocial leadership styles in contrast to their antisocial opposites will play a key role in creating compassionate social contexts and leaders (Gilbert, 1989/2016, 2009, 2018). Importantly, moral and compassionate motives and behaviors are subject to both facilitators and inhibitors, with fear of the consequences of behaving compassionately being a major inhibitor (Gilbert and Mascaro, 2017). Understanding these relationships may also have key implications.

### Possible Ways of Comparing Prosocial and Antisocial Styles

Given the processes outlined above, we have chosen a set of self-report scales that might give new insights into these dimensions of antisocial and prosocial leadership styles. As noted, Zuroff et al. (2010) described three leadership styles that they call ruthless self-advancement, dominant and coalition building. To these we can add standard measures of hypercompetitiveness (Ryckman et al., 1990) and narcissism (Ames et al., 2006) because, as noted above, these have also been identified as being linked to the dimensions of prosocial and antisocial leadership styles. While there may be differences in the nature of competitiveness between the antisocial and prosocial styles, it is unclear as to the focus of that competitiveness. Some forms of competitive striving are linked to the desire for dominance, control over others and a sense of superiority (Martin et al., 2014) and issues of greed (Van Kleef et al., 2008). Some leadership styles may also be related to the fears of inferiority and the avoidance of being marginalized, subordinated and rejected. Thus, an interesting question is: to what degree are antisocial or prosocial forms of leadership threat-driven? Gilbert et al. (2007) developed measures that distinguish between insecure and secure achievement striving. Insecure striving and competitive behavior are linked to fears of: failure, active rejection, being passed over or marginalized, losing out/missing out on opportunities, depression and anxiety. In contrast, secure striving was not linked to the fear of failure, nor to worries of rejection in the face of failing. The study found that insecure striving was associated with hypercompetitive attitudes (*r* = 0.57) and insecure attachment (*r* = 0.56) (Gilbert et al., 2007). While these processes have been examined in relation to depression and anxiety, they have not been explored in relation to different leadership styles.

One central domain of prosocial leadership is compassion. Therefore we sought to explore different dimensions of compassion in relation to the other variables. Crocker and Canevello (2008) developed a measure to distinguish between prosocial and compassion focused motives and ego focused competitive and shame avoidant motives (wanting to be recognized and to avoid being seen as wrong). Compassion and prosocial motives were correlated with increased social support, reduced loneliness and better wellbeing, whereas ego focused shame avoidance was correlated with conflict, increased loneliness and poorer mental health. Another dimension of compassion is that people can be fearful and resistant to it. A number of studies have shown that fears of compassion to others, fears of compassion from others, and fear of compassion to oneself are associated with a range of mental health problems (Gilbert et al., 2011). We wanted to explore if compassion and fears of compassion would map onto antisocial and prosocial leadership styles.

Finally, we note that attachment style has been identified as a theme that may underpin prosocial and antisocial leadership styles. Zuroff et al. (2010) found that the ruthless advancement style was linked to insecure and unsafe attachment styles and in particular avoidant attachment. Linked to attachment style is the degree to which forms of leadership feel relatively safe or threatened in their social environments. Kelly et al. (2012) showed that feeling socially safe and connected with others were better predictors of psychopathology than positive and negative affect and social support. It is unclear the degree to which antisocial styles of leadership do or do not feel socially connected and safe in their social contexts.

### Study: Aims and Objectives

The aims of this study were to explore how a set of established self-report questionnaires might relate to dimensions of prosocial and antisocial leadership styles. We hypothesized that antisocial leadership styles would correlate with, hypercompetitive attitudes, narcissism, insecure striving (striving to avoid inferiority and marginalization), fearfulness of compassion, being avoidant in their attachment style and feeling relatively socially unsafe. In contrast, we hypothesized that the prosocial leadership style would show the opposite pattern. In essence, we propose that the antisocial leadership style is more competitive insecure, threat and self-focused, whereas the prosocial style is more compassion focused and secure.

Given that compassion may be a central dimension differentiating these two leadership styles, we explored possible mediation effects of fears of compassion on the relationship with study variables.

## Materials and Methods

### Participants and Procedure

The study was approved by the Psychology Research Ethics Committee at the University of Derby. It was conducted in 2016 for 3 months. All participants (*N* = 219) read an information pack, provided written consent in accordance with the Declaration of Helsinki (2013) Ethical Principles for Medical Research involving Human Subjects, and completed the study questionnaire pack on paper or online via Qualtrics^1^ (Qualtrics, Provo, UT, United States; LimeSurvey Project Team/Carsten Schmitz, 2015). The students had as much time as they required to fill in the questionnaires. All participants were provided with a debriefing sheet and £5 gift voucher for their participation.

Four students were statistically identified by *z*-scores as outliers, in more than one variable, and were removed from the dataset, leaving *N* = 219. The final sample comprised of 139 females and 80 males with ages (two participants had missing age information) ranging from 18 to 49 years (*M* = 24, *SD* = 6.05).

### Measures

#### Demographics Form

All participants completed a demographic form including items such as gender, age, and their degree course (e.g., Business, Psychology, Health and Social Care, Science, Law, Humanities and Social Sciences, Art, Education or Other).

#### Narcissistic Personality Inventory (NPI-16)

The NPI-16 (Ames et al., 2006) 16-item scale was constructed from the original Raskin and Terry (1988) 40-item measure. Participants are given a pair of statements and asked to identify the one that best describes their feeling and beliefs about themselves. For example, “I really like to be the center of attention” and “It makes me uncomfortable to be the center of attention.” The NPI-16 has good reliability with a Cronbach’s alpha of 0.72 (Ames et al., 2006).

#### Friendship Compassionate, and Self-Image Goals

This 13-item scale developed by Crocker and Canevello (2008) has two subscales: seven items assessing compassionate goals and six items assessing self-image goals. All items began with, “In the past week, in the area of friendships, how much did you want to or try to….” The items are rated on a scale ranging from 1 (not at all) to 5 (always). The subscales have good reliability, with a Cronbach’s alpha of 0.83 for the self-image goals and of 0.90 of for the compassionate goals (Crocker and Canevello, 2008).

#### Striving to Avoid Inferiority Scale (SAIS)

The scale was designed by Gilbert et al. (2007) and has two parts. Part 1 has 31 items, which measure beliefs about striving to compete to avoid inferiority and feelings of acceptance or rejection by others if one fails. There are two factors, “insecure striving” and “secure non-striving.” All items are answered using a five-point Likert scale of 0 = never to 4 = always.

The second part of the SAIS focuses on reasons for insecure striving, which are fears of losing out, being overlooked (which was regarded as a form of passive exclusion) and active rejection (involves being shamed and pushed away). Participants respond to statements on a 10-point scale, ranging from 1 = “don’t agree” to 10 = “completely agree.” Both parts of the SAIS have good reliability, with Cronbach’s alphas of 0.84 for insecure striving, 0.69 for secure non-striving, 0.84 for losing out, 0.80 for being overlooked and 0.79 for rejection (Gilbert et al., 2007).

#### Experiences in Close Relationships Scale

The 36-item Experiences in Close Relationships scale was designed by Brennan et al. (1998). It was designed to measure attachment-related avoidance (e.g., “I get uncomfortable when a romantic partner wants to be very close”) and anxiety (e.g., “I worry about being abandoned”). For the instructions, participants are asked to think about their close relationships without focusing on a specific partner and to rate the extent to which each item describes their feelings using a seven-point scale, ranging from 1 (not at all) to 7 (very much). The subscales have good reliability with Cronbach’s alphas of 0.91 for anxiety and 0.94 for avoidance (Brennan et al., 1998).

#### Social Safeness and Pleasure (SSPS) Scale

The SSPS (Gilbert et al., 2009) assesses the extent to which individuals feel a sense of warmth, acceptance, and connectedness in their social world. Participants rate each item on a Likert scale from 1 (“almost never”) to 5 (“almost all the time”). Kelly et al. (2012) found that social safeness was more strongly correlated with vulnerability and psychopathology than negative affect, positive affect and perceived social support. This study will be the first to explore how different types of competitive behavior are linked to perceptions of social connectedness. The scale has good reliability, with a Cronbach’s alpha of 0.96 (Kelly et al., 2012).

#### Hypercompetitive Attitudes (HCA) Scale

The 26-item scale from Ryckman et al. (1990) is based on Horney (1937) 90-item scale developed to measure hypercompetitiveness. Items are rated on a 5-point scale (from 1 = Never true of me to 5 = Always true of me). There are hypercompetitive items (e.g., “Winning in competition makes me feel more powerful as a person”) and reverse-scored non-hypercompetitive items (e.g., “I do not see my opponents in competition as my enemies”). The scale has good reliability, with a Cronbach’s alpha of 0.91 and 6-week test-retest reliability and convergent validity (Ryckman et al., 1990).

#### The Rank Style With Peers Questionnaire (RSPQ)

The RSPQ is a 17-item scale developed by Zuroff et al. (2010) and comprises three subscales: dominant leadership, coalition building and ruthless self-advancement. A series of studies demonstrated that the RSPQ’s factor structure is robust and is a reliable measure (Kelly et al., 2011; Zuroff et al., 2010).

### Fears of Compassion

This 38-item scale has three subscales: fears of expressing compassion for others (10 items, e.g., “Being too compassionate makes people soft and easy to take advantage of”); fears of receiving compassion from others (13 items, e.g., “I try to keep my distance from others even if I know they are kind”); and fears of compassion for self (15 items, e.g., “I worry that if I start to develop compassion for myself, I will become dependent on it”). Participants rate on a Likert scale how much they agree with each statement, from 0 = Don’t agree at all, to 4 = Completely agree. In a student sample, Gilbert et al. (2011) reported Cronbach’s alphas as 0.72 for fears of expressing compassion for others, 0.80 for fears of receiving compassion from others, and 0.83 for fears of giving compassion to self. Importantly, since the development of the scale it’s become clear that the items in the ‘fears of compassion to others’ scale may be tapping more resistance than fears as such. So it may be better to interpret the results in terms of compassion resistance–even though such resistance may ultimately rest on underlying fears.

### Data Analyses

Data analyses were conducted using Predictive Analytics Software (PASW, version 23) and Analysis of Momentary Structure (AMOS; Spss Inc., Released, 2009). Data was checked for normality and outliers. Skewness values ranged from 0.01 to 0.87 and kurtosis values ranged from 0.03 to 0.84, which is within acceptable ranges (Kline, 2005). Four participants were identified statistically as outliers in more than one variable and were removed from the dataset, leaving *N* = 219.

A series of Pearson product moment correlation analyses were performed to assess the relationship between all variables. Following this, a principal component analysis was conducted with all variables included. The results indicated six possible factors. These were difficult to interpret and had a number of cross-loadings. Given our intention to explore an antisocial dimension and the relationships observed in the correlation analyses, ruthless self-advancement, narcissism and hypercompetitive attitude variables were entered into a principal component analysis (Maximum Likelihood extraction). The Kaiser-Meyer-Olkin and Bartlett’s Test of Sphericity both indicated the sample size was adequate for a principal component analysis (Field, 2005). Results of the principal component analysis did suggest a single factor, which we labeled antisocial leadership style.

We then conducted a series of Pearson product moment correlation analyses to assess the relationship between this new variable of antisocial leadership style with dominant leadership style and coalition building leadership style; fears of compassion for others, from others and for self; self-image goals and compassionate goals; insecure striving and secure non-striving; fears of losing out, being over looked and active rejection; attachment anxiety and attachment avoidance; and social safeness.

Moreover, a multiple linear regression (Enter Method) with the antisocial leadership factor as dependent variable and fears of compassion for others, from others, and for self, insecure striving, fears of losing out, being overlooked, active rejection, avoidance in close relationships, and self-image goals as independent variables was conducted. Analysis of histograms, P-P plots, regression plots of standardized residuals against standardized predicted values showed that assumptions of linearity and normality of distribution were met. There was no evidence of multicollinearity (i.e., tolerance >0.20 and VIF <5.00, Field, 2005).

Path analyses were conducted to test for mediator effects. Path analysis is a special case of Structural Equation Modeling and considers hypothetical causal relations between variables that have already been defined. The SEM procedure estimates the optimal effect of one set of variables on another set of variables in the same equation, controlling for error (Kline, 2005; Byrne, 2010). We generated two models. One was focused on the new variable, antisocial leadership style (based on composite score). The second was based on the prosocial leadership style, here we used one variable which was the coalition building style. From the multiple regression, those variables that had predictive power on antisocial leadership style were entered as independent, exogenous variables with the antisocial leadership style as the dependent, endogenous variable (Model A). We then used avoidant attachement, secure non-striving and compassionate goals variables as independent, exogenous variables in Model B, with the coalition building leadership style as the dependent, endogenous variable.

The significance of the regression coefficients and the fit statistics were tested using the Maximum Likelihood estimation method. Multivariate outliers were screened using the Mahalanobis squared distance (D2) method and univariate and multivariate normality was assessed by skewness and kurtosis coefficients and found to be acceptable. The following indices were selected to examine model fit (Kline, 2005): Normed Chi-square (χ^2^/df), with 2–5 indicating good fit;, Comparative Fit Index (CFI), Tucker-Lewis Index (TLI), and Bentler-Bonett or Normed Fit Index (NFI) with values above 0.90 suggesting good fit;, and Root Mean Square Error of Approximation (RMSEA), with 0.05–0.08 indicating reasonable error and acceptable fit. The significance of direct, indirect and total effects was assessed using χ^2^ tests. A Bootstrapping resampling method was further used to test the significance of the mediational paths, using 5000 bootstrap samples and 95% confidence intervals (CIs; Kline, 2005).

## Results

Means, standard deviations, and Cronbach’s alphas of all variables are presented in Table 1.

**Table 1:** Means and standard deviation of all study variables.

	Total *N* = 219 M (*SD*)	Cronbach’s alpha
		
RSPQ		
Ruthless self-advancement	13.30 (5.11)	0.86
Dominant leadership	17.34 (4.90)	0.91
Coalition building	28.51 (4.90)	0.86
Narcissism	0.21 (0.19)	0.77
Hypercompetitive attitudes	69.63 (13.14)	0.80
SAIS		
Insecure striving	38.39 (14.63)	0.92
Secure non-striving	29.79 (8.69)	0.88
Fear of losing out	18.06 (6.83)	0.89
Fear of overlooked	22.15 (8.94)	0.93
Fear of active rejection	17.42 (9.07)	0.93
ECR		
Avoidance	57.84 (23.33)	0.93
Anxiety	62.57 (23.03)	0.92
SSPS	38.15 (9.67)	0.94
Fears of compassion		
For others	16.72 (8.70)	0.88
From others	15.24 (10.30)	0.90
For self	14.89 (12.97)	0.94
FCSIGS		
Compassionate goals	24.42 (5.08)	0.77
Self-image goals	16.40 (5.21)	0.82


*RSPQ, Rank Style with Peers Questionnaire, Ruthless self-advancement, Dominant leadership, Coalition building; Narcissism, Narcissistic Personality Inventory-16; Hypercompetitive Attitudes, Hypercompetitive Attitudes Scale; SAIS, Striving to avoid inferiority scale: Insecure striving, secure non-striving, fear of losing out, fear of overlooked, fear of active rejection; ECR, Experience in close relationships: avoidance and anxiety; SSPS, Social safeness and pleasure scale; Fears of compassion, fears of compassion scale, for others, from others, for self; FCSIGS, Friendship compassionate and self-image goals scale, compassionate goals, self-image goals.*

To assess the relationship between all variables, Pearson product-moment correlation analysis was performed and is given in Table 2.

**Table 2:** Pearson’s correlations between different styles of leadership, striving to avoid inferiority, fears of compassion, and attachment security.

	1	2	3	4	5	6	7	8	9	10	11	12	13	14	15	16	17
(1) Ruthless self advancement leadership style																	
(2) Dominant leadership style	0.31**																
(3) Coalition building leadership style	–0.17*	0.38**															
(4) Narcissism	0.41**	0.38**	–0.24**														
(5) Hyper-competitiveness	0.50**	0.15*	–0.31**	0.31**													
(6) Fears of compassion: for others	0.33**	0.09	–0.21**	0.23**	0.35**												
(7) Fears of compassion: from others	0.32**	0.02	–0.22**	0.19**	0.29**	0.60**											
(8) Fears of compassion: for self	0.37**	0.02	–0.29**	0.18**	0.26**	0.48**	0.75**										
(9) Self-image goals	0.32**	0.05	–0.24**	0.21**	0.38**	0.28**	0.36**	0.38**									
(10) Compassionate goals	–0.19**	0.17*	0.24**	–0.14*	–0.27**	–0.16*	–0.15*	–0.05	0.26**								
(11) Insecure striving	0.46**	0.16*	–0.13	0.24**	0.55**	0.46**	0.46**	0.48**	0.50**	–0.09							
(12) Secure non-striving	–0.07	0.11	0.15*	0.02	–0.24**	–0.09	–0.34**	–0.33**	–0.18**	0.09	–0.11						
(13) Fear of losing out	0.38**	0.12	0.03	0.18**	0.44**	0.32**	0.30**	0.34**	0.44**	0.05	0.55**	–0.21**					
(14) Fear of overlooked	0.43**	0.12	–0.12	0.21**	0.47**	0.32**	0.39**	0.37**	0.44**	0.01	0.60**	–0.22**	0.65**				
(15) Fear of active rejection	0.38**	0.02	–0.24**	0.15*	0.41**	0.35**	0.39**	0.40**	0.41**	–0.04	0.55**	–0.27**	0.49**	0.71**			
(16) ECR: anxiety	0.09	–0.08	0.03	–0.06	0.07	0.22**	0.27**	0.21**	0.31**	0.21**	0.28**	–0.07	0.32**	0.28**	0.16*		
(17) ECR: avoidance	0.32**	0.03	–0.27**	0.20**	0.23**	0.23**	0.47**	0.47**	0.23**	–0.12	0.27**	–0.30**	0.18**	0.29**	0.23**	0.02	
(18) Social safeness	–0.05	0.08	0.22**	–0.05	–0.20**	–0.30**	–0.55**	–0.47**	–0.20**	0.16*	–0.30**	0.42**	–0.19**	–0.23**	–0.24**	–0.13	–0.48**


*^∗∗^Correlation is significant at the 0.001 level (2-tailed); ^∗^Correlation is significant at the 0.05 level (2-tailed). (1–3) RSPQ, Rank Style with Peers Questionnaire: Ruthless self-advancement, Dominant Leadership, Coalition Building; (4) Narcissism, Narcissistic Personality Inventory-16; (5) Hypercompetitive Attitudes, Hypercompetitive Attitudes Scale; (6–8) Fears of Compassion, Fears of compassion scale: for others, from others, for self; (9) FCSIGS, Friendship compassionate and self-image goals scale: compassionate goals, self-image goals; (11–15) SAIS, Striving to avoid inferiority scale: insecure striving, secure non-striving, fear of losing out, fear of overlooked, fear of active rejection; (16-17) ECR, Experience in close relationships: avoidance and anxiety; (18) SSPS, Social safeness and pleasure scale.*

Table 2 reveals a number of correlations in the expected direction. For example, ruthless self-advancement leadership is positively correlated with the dominant leadership style, hypercompetitiveness, narcissism, fears of compassion, self-image goals, various forms of insecure striving, and the avoidant relating style, whilst significantly negatively correlated with coalition building and compassion goals. In contrast, coalition building style is negatively linked to narcissism, hypercompetitiveness, fears of compassion, self-image goals and fears of active rejection and positively linked to compassion goals, secure non-striving and a sense of social safeness.

A principal component analysis (Maximum Likelihood extraction) on the ruthless self-advancement, narcissism and hypercompetitive attitude scales produced an “antisocial leadership style” single factor with an eigenvalue of 1.80, explaining 60.09% of the variance. Loadings are presented in Table 3.

**Table 3:** Factor loadings for antisocial leadership style factor.

	Factor 1
Ruthless self advancement leadership style	0.830
Narcissism	0.706
Hyper-competitiveness	0.785
Variance (%)	60.09


A Pearson product moment correlation analysis was performed with the antisocial leadership style factor along with coalition building leadership style, dominant leadership style, fears of compassion, self image and compassion goals, striving to avoid inferiority, attachment security and social safeness, as given in Table 4. The relationship between antisocial leadership style and the other variables strengthens in the predicted direction.

**Table 4:** Pearson’s correlations between different styles of leadership, striving to avoid inferiority, fears of compassion, and attachment security.

	Dominant leadership style	Coalition building leadership style	Fears of compassion: for others	Fears of compassion: from others	Fears of compassion: for self	Self-image goals	Compassionate goals	Insecure striving	Secure non-striving	Losing out	Over looked	Active rejection	ECRS: anxiety	ECRS: avoidance	Social safeness
Antisocial leadership style	0.42^∗∗^	–0.39^∗∗^	0.43^∗∗^	0.35^∗∗^	0.36^∗∗^	0.40^∗∗^	–0.24^∗∗^	0.55^∗∗^	–0.09	0.41^∗∗^	0.46^∗∗^	0.40^∗∗^	0.01	0.34^∗∗^	–0.16^∗^


*^∗∗^Correlation is significant at the 0.001 level (2-tailed); ^∗^Correlation is significant at the 0.05 level (2-tailed). Antisocial leadership style; composite score of Ruthless self-advancement, Narcissism and Hypercompetitiveness; RSPQ, Rank Style with Peers Questionnaire, Dominant Leadership, Coalition Building; Fears of Compassion, fears of compassion scale: for others, from others, for self; FCSIGS, Friendship compassionate and self-image goals scale: self-image goals, compassionate goals; SAIS, Striving to avoid inferiority scale: insecure striving, secure non-striving, fear of losing out, fear of overlooked, fear of active rejection; ECR, Experience in close relationships: avoidance and anxiety; SSPS, Social safeness and pleasure scale.*

### Multiple Regression

To explore the predictive power of the independent variables on the antisocial leadership style, we conducted a multiple linear regression (Enter Method) with the antisocial leadership factor as the dependent variable and fears of compassion for others, from others, and for self, insecure striving, fears of losing out, being overlooked, active rejection, avoidance in close relationships, and self-image goals as independent variables. The analysis accounted for 42% of the variance in the prediction of the antisocial leadership style factor [*F*(9,200) = 15.11, *p* < 0.001]. The strongest predictor was insecure striving (β = 0.310, *p* < 0.001), followed by fears of compassion for others (β = 0.213, *p* = 0.004) and avoidance in close relationships (β = 0.167, *p* = 0.01) as given in Table 5. The other independent variables were not significant predictors of antisocial leadership style.

**Table 5:** Multiple linear regression with antisocial leadership as the dependent variable and fears compassion for others, from others, and for self, insecure striving, fears of losing out overlooked, active rejection, avoidance in close relationships, and self-image goals as independent variables.

	*B*	*SE B*	β
**Constant**	–2.312	0.240	
Fears compassion for others	0.025	0.009	0.213*
Fears compassion from others	–0.007	0.010	–0.066
Fears compassion for self	–0.001	0.007	–0.014
Striving to avoid inferiority scale insecure striving	0.022	0.006	0.310**
Striving to avoid inferiority scale losing out	–0.001	0.013	–0.009
Striving to avoid inferiority scale overlooked	0.016	0.011	0.142
Striving to avoid inferiority scale active rejection	0.001	0.009	0.007
Experience in close relationships-avoidance	0.007	0.003	0.167*
Self-image goals	0.024	0.013	0.124


*R^2^ = 0.42 (p < 0.001); ^∗^p < 0.01; ^∗∗^p < 0.001.*

### Path Analysis

**FIGURE 1:**
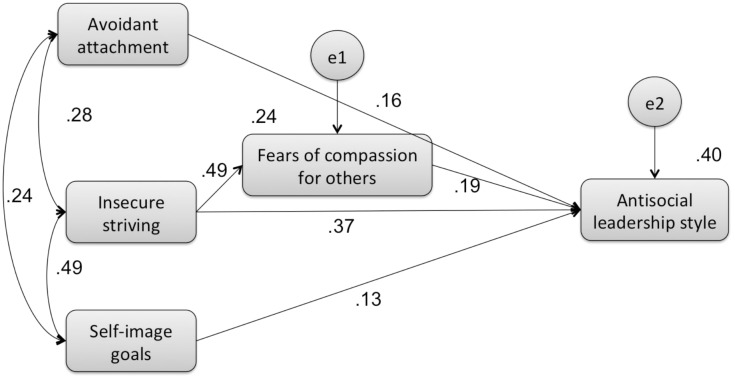
Results of the adjusted model for the mediation path analysis showing the relationships among fears of compassion for others, avoidant attachment, insecure striving, self-image goals, and antisocial leadership style.

Model A: path model of the mediator effect of fears of compassion on the relationship between avoidant attachment, insecure striving and self-image goals (exogenous variables), and the antisocial leadership style (endogenous variable) (Figure 1).

The relationships between variables generated from the multiple regression analysis were tested through a fully saturated model (i.e., zero degrees of freedom), consisting of 35 parameters. Given that fully saturated models always produce a perfect fit to the data, model fit indices were neither examined nor reported. Model A explained 40% of antisocial leadership style variance. Several paths were not statistically significant and were therefore removed. Additionally, fears of compassion for the self and from others were not significant predictors of antisocial leadership style and were therefore excluded from the final adjusted model and the model was recalculated.

The final adjusted model, consisting of 18 parameters, is depicted in Figure 1. Path analysis results showed that the model presents a very good fit to the data, χ^2^/df = 1.860, *p* = 0.156, CFI = 0.992, TLI = 0.962, NFI = 0.984, RMSEA = 0.065. All the paths were statistically significant and bootstrap resampling method results confirmed the significance of the indirect mediational paths. The final model accounted for 40% of antisocial leadership style variance.

Indirect mediational test results indicated that insecure striving predicted higher levels of the antisocial leadership style, partially through increased fears of compassion for others (β = 0.095, 95% CI = 0.048–0.152), with insecure striving still having a direct effect on the dependent variable (*b* = 0.025, SEb = 0.005; *Z* = 5.160; β = 0.366, *p* < 0.001). Both avoidant attachment (*b* = 0.007, SEb = 0.002; *Z* = 2.699; β = 0.155, *p* = 0.007) and self-image goals (*b* = 0.026, SEb = 0.012; *Z* = 2.131; β = 0.135, *p* = 0.033) directly predicted antisocial leadership style and the fears of compassion for others did not emerge as a significant mediator.

These findings reveal that fears of compassion for others is the only significant partial mediator between insecure striving and antisocial leadership style. Fears of compassion for others does not mediate the relationship between avoidant attachment and self-image goals with antisocial leadership style.

**FIGURE 2:**
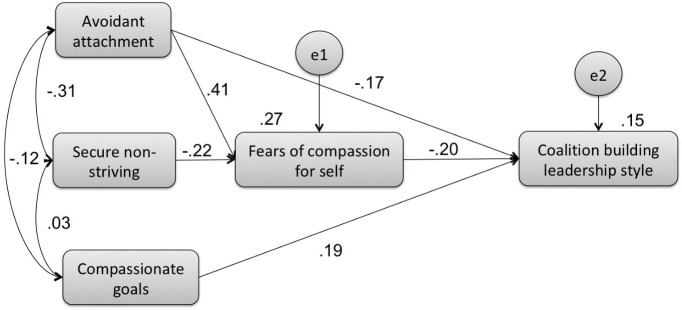
Results of the adjusted model for the mediation path analysis showing the relationships among fears of compassion for self, avoidant attachment, secure non-striving, compassionate goals, and coalition building leadership style.

Model B: Path model of the mediator effect of fears of compassion on the relationship between avoidant attachment, secure non-striving and compassionate goals (exogenous variables), and the coalition building leadership style (endogenous variable) (Figure 2).

Avoidant attachment, secure non-striving and compassionate goals were entered as independent variables, but with the coalition building style as the dependent variable for the analysis. It was tested utilizing a fully saturated model consisting of 35 parameters. The model explained 17% of coalition building leadership style variance. Several paths were not statistically significant and were therefore removed. In this model, fears of compassion for others and from others were not significant predictors of the coalition building leadership style and were therefore excluded from the final adjusted model and the model was recalculated.

The final adjusted model, consisting of 18 parameters, is depicted in Figure 2. Results showed that the model presents an excellent fit to the data, χ^2^/df = 0.099, *p* = 0.906, CFI = 1.000, TLI = 1.082, NFI = 0.998, RMSEA = 0.000. This final model accounted for 15% of coalition building leadership style variance and all the paths were statistically significant, with bootstrap resampling method results confirming the significance of the indirect mediational paths.

Indirect mediational test results indicated that secure non-striving predicted higher levels of the coalition building leadership style fully through decreased fears of compassion for self (β = 0.044, 95% CI = 0.014–0.095). Avoidant attachment predicted decreased levels of the coalition building leadership style, partially through increased fears of self-compassion (β = -0.083, 95% CI = -0.149 to -0.031), while still having a significant direct effect on this dependent variable (*b* = -0.034, SEb = 0.015; *Z* = -2.273; β = -0.168, *p* = 0.023). Compassionate goals (*b* = 0.189, SEb = 0.064; *Z* = 2.953; β = 0.193, *p* = 0.003) had a direct effect on the coalition building leadership style and the fears of compassion for self did not emerge as a significant mediator.

Results indicated that, for coalition building leadership style, fears of compassion for self is a significant mediator. It fully mediates the relationship between secure non-striving and coalition building leadership style and it partially mediates the impact of avoidant attachment on coalition building leadership style. Fears of compassion for self does not mediate the relationship between compassionate goals and coalition building leadership style.

## Discussion

This study set out to explore two types of leadership styles, labeled antisocial and prosocial, linked to different evolutionary survival and reproductive strategies (Brañas-Garza et al., 2016; Ewest, 2017; Gilbert, 2018; Gilbert and Basran, 2018). Whereas prosocial leadership styles focus on the needs and difficulties of others, with an intention to be helpful rather than harmful, antisocial leadership styles have a callous indifference to the needs and difficulties of others and are not averse to being harmful if it advances them or defends their position. Presently, there is no consensus on the “best” measures to tap into these styles, but various dimensions of personality, such as the dark triad (Furnham et al., 2013) and social dominance orientation (Ho and Sidanius, 2010), maybe typical elements of the antisocial leadership style.

In this study, we used a different set of measures, as outlined above, to explore how a set of established self-report questionnaires might relate to dimensions of antisocial and prosocial leadership styles. We conducted a principal component analysis with all measures included, which indicated six possible factors. However, they were difficult to interpret in light of previous literature, and had a number of cross-loadings. It was clear, however, that one set of variables did cluster together, namely ruthless self-advancement, hypercompetitiveness and narcissism. A principal component analysis with these variables included confirmed this factor structure and, we named this antisocial leadership style. As dominant leadership style was positively related to both coalition building style and ruthless self-advancement, this was not included in the analysis. The principal component analysis did not suggest a single such factor for the prosocial leadership style. So, on this occasion, we used the single variable of coalition building leadership style.

### Antisocial Leadership Style

Using the composite score labeled antisocial leadership style, we explored specific correlations with this variable (Table 4). The antisocial leadership style interestingly has some overlap with the dominant leadership style whereas, the coalition building style is negatively correlated with the antisocial leadership style. Looking at the other variables, antisocial leadership style is linked to fears of compassion, particularly being fearful of offering compassion to others. In our method sections we noted that the fears of compassion to others is tapping more resistance themes than fears as such. So it may be better to interpret what has been called fears’ more in terms of resistance to compassion. It is also associated with ego goals and negatively associated with compassion goals. Hence, both the fears of compassion scale and the measure of compassion and ego goals scale tell the same story, that these individuals are avoidant of and resistant to being helpful to others, maybe indicating a heightened self-concerned focus.

There are many reasons that people will compete for social place, achievements and resources. One of them is threat-based, to avoid being rendered inferior, marginalized or actively rejected. Importantly, we found that there was a strong correlation between antisocial leadership style and striving to avoid inferiority (insecure striving) and its associated fears of losing out, being overlooked and active rejection. In a highly competitive environment, these outcomes could have serious consequences.

As to the general feelings of social safeness, there was a small negative correlation with antisocial leadership, suggesting that these individuals do not feel particularly safe, although the correlation is small. This could be because, individuals with narcissistic tendencies may assume that others accept and like them, perhaps a sort of grandiose feeling of connectedness, but it might be fragile.

The results also suggest that the antisocial leadership style is associated with avoidant attachment. Zuroff et al. (2010) also found ruthless self advancement was associated with avoidant attachment, indicating these individuals struggle with emotional closeness. This has been explored within the attachment theory literature too, with concern being raised that people who are avoidant in attachments tend to seek out positions of power and can be callous (Mikulincer and Shaver, 2007, 2017). It would seem then, that this leadership style fits with other elements of the literature, whereby some individuals see the world as a threatening place, a “dog-eat-dog world,” where you do not go out of your way to help others (Janoff-Bulman, 2009).

### Prosocial Leadership Style

As suggested above, we did not develop a composite score for the prosocial leadership style, but refer primarily to the style of coalition building (Zuroff et al., 2010). Table 2 shows that this style of leadership is associated with similar variables as the antisocial leadership style, but completely in the opposite direction. Coalition building is negatively associated with the ruthless self-advancement style, dominant leadership style, narcissism, hypercompetitiveness, fears of compassion for others, fears of compassion from others and fears of compassion for self, self-image goals, fears of rejection and avoidant attachment. It is positively correlated with secure striving, compassionate goals and feeling socially safe. In comparison with the antisocial leadership style, where there are clear associations between this leadership style and other variables measured, for coalition building or the prosocial leadership style, these are much weaker. This suggests that there is considerable variation between those who adopt this leadership style.

### Multiple Regression and Path Analysis

We then explored the main predictors of antisocial leadership style in a multiple regression (Table 5). This revealed that insecure striving, fears of compassion for others and avoidance in close relationships predict the antisocial leadership style.

Furthermore, the results of the path analysis with antisocial leadership style (Figure 1) suggest that fears of compassion for others partially mediates the relationship between antisocial leadership style and insecure striving. The threat-based need to succeed in the world, associated with not wanting to help others, seems particularly pertinent to antisocial leadership style. Avoidant attachment and self-image goals have direct effects on antisocial leadership style and fears of compassion for others was not a significant mediator.

In Figure 2, we explored similar relationships, but with coalition building leadership style. Here, lower fears of compassion for self fully mediated the relationship between secure non-striving and increased levels of the coalition building style. Increased fears of compassion for self partially mediated the relationship between avoidant attachment and decreased levels of the coalition building leadership style. Compassionate goals had a direct effect on coalition building style and fears of compassion for self was not a significant mediator. Taken together, this indicates that individuals who are secure with close attachments and feel secure in their striving (rather than feeling worried about being rejected, criticized or failing) and are able to be compassionate and supportive to themselves, are presumably capable of creating prosocial styles of leadership in teams they lead.

Taken together, the results are consistent with previous studies in that we can identify two different dimensions of leadership. One is threat and self-focused competitive, which is compassion-resistant and attachment-avoidant. It is a style that is underpinned by the fear of being inferior, being rejected, overlooked and missing out on opportunities. In contrast, the other is underpinned by individuals who feel relatively safe with others, are more likely to be compassionate to themselves, have compassion goals and feel relatively secure in their competitive behavior.

There are a number of possible reasons why no clear set of measures provided a coherent factor for the prosocial leadership style, unlike the antisocial leadership style. The most obvious is that we did not have the appropriate measures to tap it. Second is the possibility that the prosocial leadership style is far more variant than the antisocial style. This is partly because the antisocial leadership style seems so orientated around threat, whereas prosocials may have a number of different goals and issues. For example, some individuals may not be antisocial, but their leadership style may be more of a career path and they are interested in doing their jobs, but not necessarily being particularly helpful to others. It is likely there is a leadership style that is pragmatic, rather than particularly antisocial or prosocial. In terms of leadership training, clearly those who adopt prosocial and antisocial leadership styles will need very different orientations in training. For example, antisocial leaders are highly threat-focused and if that is not addressed, then they may have little chance of becoming more altruistic and prosocial. Interestingly, Bargh (2017), reviews a number of studies showing that right-wing individuals see the world in a much more threatening way than left-wing individuals. However, the safer and more connected you can help these individuals feel, the more likely they are to have values of sharing and helpfulness.

### Limitations

As this is a cross-sectional study conducted with a student population, issues of generalizability arise. Having illustrated these connections, subsequent studies need to look at individuals in leadership positions. We suspect that the relationships will be even stronger. Here we refer to *leadership style*, not leadership, as we were interested in the manner and approach participants adopt to manage people. We do not know how contextual these styles are; for example, is the bullying, hard driving boss at work the same with friends or family or even different types of working environment? We also have to raise the issue of measures. For example, new measures could be generated specifically designed to compare and contrast prosocial and antisocial leadership rather than relying on measures that were designed for other tasks. Currently, we did not obtain test-retest reliability for these traits, therefore we cannot say how stable these are, but we have no reason to believe they would not be stable. We have not explored issues of gender variation, which may play a role. This study has a higher percentage of females than males 139/80. It’s possible that these dimensions are influenced by gender which further research may illuminate.

Many other variables that are clearly important in relation to leadership style that are not part of this study, such as capacities for empathy, emotion regulation and moral reasoning, would be candidates for future studies. Contexts in which people operate may also be areas of further investigation, as to assess whether those contexts encourage or discourage prosocial or antisocial leadership styles. In this study, we had not controlled for this, partly because this is a student population, but subsequent studies with people in managerial and leadership positions should explore the degree to which they perceive their organization as encouraging or discouraging prosocial and antisocial leadership. Finally we note that the boundaries between resistance to ‘compassion to others’ and (possible underlying) fears for that resistance is unclear. Future research would need to clarify this distinction better than we could here.

## Conclusion

At its simplest, it seems that the antisocial leadership style is basically a threat and self-focused competitive orientation to the challenges of competing for resources and finding status and social position in the world. It is not surprising then that there are often leaders who utilize the language of threat to generate support or subdue dissent and are callous to the harm they may cause. History shows they are often power-orientated and have very destructive impacts on the groups they lead (Gilbert, 2018). Clearly, if we are to promote a more compassionate and socially fair world, the dynamics of these styles of leadership and how to promote them need to be better understood. It would also be necessary to learn how to ensure prosocial styles are attractive to those who might vote for them or follow them. These are fundamental to developing the moral and ethical societies that many seek to create (Hannah et al., 2011).

## Ethics Statement

All procedures received approval by the Psychology Research Ethics Committee at the University of Derby. Students were asked to complete questionnaires either on paper at the end of their lectures or during lecture breaks or online via online tools [www.qualtrics.com; Qualtrics, Provo, UT, United States; LimeSurvey Project Team/Carsten Schmitz (2015)] which produced a SPSS data output file downloaded by the researcher upon the completion of data collection. All participants read an information pack, provided written consent, and filled out the study questionnaire pack. All subjects gave written informed consent in accordance with the Declaration of Helsinki. They were subsequently given a debriefing sheet and £5 gift voucher for their participation.

## Author Contributions

PG and JB were involved in all aspects of the study. CP collected and inputted it the data into databases. MM and KM analyzed and interpreted the data.

## Conflict of Interest Statement

The authors declare that the research was conducted in the absence of any commercial or financial relationships that could be construed as a potential conflict of interest.

## Footnotes

1 www.qualtrics.com
